# Immune response from STRIDE, a randomized, Phase II, open-label study of sipuleucel-T (sip-T) with concurrent vs sequential enzalutamide (enz) administration in metastatic castration-resistant prostate cancer (mCRPC)

**DOI:** 10.1186/2051-1426-3-S2-P145

**Published:** 2015-11-04

**Authors:** Charles G Drake, David Quinn, Robert Dreicer, Emmanuel Antonarakis, Neal Shore, John Corman, Raoul Concepcion, Christopher Pieczonka, Dwayne Campogan, Li-Qun Fan, Nancy Chang, Nadeem Sheikh, Daniel Petrylak

**Affiliations:** 1 Department of Oncology, Sidney Kimmel Comprehensive Cancer Center at Johns Hopkins University, Baltimore, MD, USA; 2 Norris Comprehensive Cancer Center, University of Southern California, Los Angeles, CA, USA; 3 University of Virginia Cancer Center, Charlottesville, VA, USA; 4 Johns Hopkins University School of Medicine, Baltimore, MD, USA; 5 Atlantic Urology Clinics, Myrtle Beach, SC, USA; 6 Virginia Mason Cancer Institute, Seattle, WA, USA; 7 Urology Associates, Nashville, TN, USA; 8 Associated Medical Professionals of New York, Syracuse, NY, USA; 9 Dendreon, Seattle, WA, USA; 10 Yale Cancer Center, New Haven, CT, USA

## Background

P12-2 (STRIDE; NCT01981122) is an ongoing, randomized, Phase II, open-label study evaluating concurrent vs sequential administration of the androgen receptor inhibitor, enz, with the autologous cellular immunotherapy, sip-T. The primary aim of this study is to determine if the order of sip-T and enz administration impacts immune responses to the immunizing antigen, PA2024, of sip-T.

## Methods

Patients with asymptomatic or minimally symptomatic mCRPC were randomized 1:1 to receive 3 sip-T infusions with enz starting 2 weeks (wks) before (n=25, concurrent arm A) or 10 wks after (n=27, sequential arm B) sip-T initiation. Antigen-specific cellular and humoral immune responses were evaluated via interferon (IFN)-γ ELISPOT, cell proliferation, and antibody ELISA assays. In addition, the breadth of the humoral response to non-target antigens was also studied.

## Results

T cell proliferative responses to PA2024 were significantly elevated at all post-baseline time points (p < 0.001) and were sustained through wk 26, including a >10-fold increase at wk 20 in both arms. Both arms showed a significant and sustained increase in IFN-γ ELISPOT response to PA2024 and humoral responses to PA2024 and PAP. Broadening of the humoral responses to non-target antigens PSA, LGALS3, LGALS8, KRAS, ERAS and KLK2 at all post-treatment time points was observed in both arms. Cytokines indicative of immune activation (including IFN-γ, interleukin-2, and tumor necrosis factor-α) were also elevated in both arms, at the 2^nd^ and 3^rd^ sip-T infusions. Adverse events were similar between arms.

## Conclusions

Treatment of mCRPC subjects with enz administered concurrently with or subsequent to sip-T resulted in significant and sustained peripheral antigen-specific T cell and humoral immune responses through wk 26. Both treatment schedules led to similarly robust humoral antigen spread sustained through wk 26. These data suggest enz did not affect sip-T production, subsequent immune responses, or safety.

## Trial registration

ClinicalTrials.gov identifier NCT01981122.

